# Effects of Neighborhood-Scale Acaricidal Treatments on Infection Prevalence of Blacklegged Ticks (*Ixodes scapularis*) with Three Zoonotic Pathogens

**DOI:** 10.3390/pathogens12020172

**Published:** 2023-01-21

**Authors:** Richard S. Ostfeld, Sahar Adish, Stacy Mowry, William Bremer, Shannon Duerr, Andrew S. Evans, Ilya R. Fischhoff, Fiona Keating, Jennifer Pendleton, Ashley Pfister, Marissa Teator, Felicia Keesing

**Affiliations:** 1 Cary Institute of Ecosystem Studies, Millbrook, NY 12545, USA; 2 Department of Behavioral and Community Health, Dutchess County, Poughkeepsie, NY 12601, USA; 3 Department of Biology, Bard College, Annandale, NY 12504, USA

**Keywords:** anaplasmosis, babesiosis, Lyme disease, tick control, tick-borne disease, zoonotic disease

## Abstract

Acaricides are hypothesized to reduce human risk of exposure to tick-borne pathogens by decreasing the abundance and/or infection prevalence of the ticks that serve as vectors for the pathogens. Acaricides targeted at reservoir hosts such as small mammals are expected to reduce infection prevalence in ticks by preventing their acquisition of zoonotic pathogens. By reducing tick abundance, reservoir-targeted or broadcast acaricides could reduce tick infection prevalence by interrupting transmission cycles between ticks and their hosts. Using an acaricide targeted at small-mammal hosts (TCS bait boxes) and one sprayed on low vegetation (Met52 fungal biocide), we tested the hypotheses that infection prevalence of blacklegged ticks with zoonotic pathogens would be more strongly diminished by TCS bait boxes, and that any effects of both acaricidal treatments would increase during the four years of deployment. We used a masked, placebo-controlled design in 24 residential neighborhoods in Dutchess County, New York. Analyzing prevalence of infection with *Borrelia burgdorferi*, *Anaplasma phagocytophilum*, and *Babesia microti* in 5380 nymphal *Ixodes scapularis* ticks, we found little support for either hypothesis. TCS bait boxes did not reduce infection prevalence with any of the three pathogens compared to placebo controls. Met52 was associated with lower infection prevalence with *B. burgdorferi* compared to placebo controls but had no effect on prevalence of infection with the other two pathogens. Although significant effects of year on infection prevalence of all three pathogens were detected, hypothesized cumulative reductions in prevalence were observed only for *B. burgdorferi*. We conclude that reservoir-targeted and broadcast acaricides might not generally disrupt pathogen transmission between reservoir hosts and tick vectors or reduce human risk of exposure to tick-borne pathogens.

## 1. Introduction

The growing prevalence of tick-borne diseases in much of the northern hemisphere has stimulated efforts to prevent human exposure by controlling tick populations [1,2]. The blacklegged tick, *Ixodes scapularis*, is the primary vector for the great majority of the tick-borne pathogens causing these diseases in the United States and Canada. Reducing the abundance of host-seeking ticks is considered one of the mainstays of disease prevention, along with modifying human behavior to reduce encounter rates with ticks and increase early detection of attached or attaching ticks [2]. Deployment of chemical and biological acaricides has been demonstrated in laboratory studies to increase mortality of blacklegged ticks [3,4] and in field studies to decrease tick abundance compared to relevant controls [5,6]. However, evidence that acaricide-reduced abundance of blacklegged ticks results in lower rates of encounters between people and ticks is weak or absent [5,6].

Pathogen transmission from a tick to a person is only possible if the tick is infected. For the major zoonotic pathogens transmitted by *I. scapularis* in North America, prevalence of infection in the nymph stage is generally <40% (e.g., for the agent of Lyme disease, *Borrelia burgdorferi*) or <15% (e.g., for the agents of human granulocytic anaplasmosis, *Anaplasma phagocytophilum*, and human babesiosis, *Babesia microti*) [7,8,9]. Hence, many human–tick encounters are not likely to result in pathogen transmission and resulting disease. Reducing the prevalence of infection of ticks with tick-borne pathogens could potentially reduce the probability of transmission to people even further.

Biological or chemical acaricides intended to reduce tick abundance could also affect tick infection prevalence. The most obvious mechanism by which tick-control can reduce tick infection is the targeting of certain host species, or groups of species, for deployment of the acaricides. Hosts vary enormously in the probability that they will transmit zoonotic infections to feeding ticks (i.e., their “reservoir competence” [10,11]). For example, ~90% of larval blacklegged ticks (which are uninfected at hatching) that feed on free-ranging white-footed mice (*Peromyscus leucopus*) acquire infections with *B. burgdorferi*, whereas <5% of those feeding on opossums (*Didelphis virginiana*), raccoons (*Procyon lotor*), or white-tailed deer (*Odocoileus virginianus*) acquire infection [11,12]. Similar variation between species of hosts in reservoir competence for *A. phagocytophilum* and *B. microti* are also observed [13,14,15]. For all three of these zoonotic pathogens, small rodents and shrews are the most competent reservoirs [7,14,15]. When acaricides are deployed in devices that target small-mammal hosts, the killing of ticks that would have fed on these hosts is expected to reduce infection prevalence in the ticks later sampled from the questing population.

A reduction in tick abundance could also potentially reduce tick infection as a result of altered transmission dynamics between hosts and ticks. If reduced abundance of host-seeking ticks reduces the rate of tick-to-host transmission of pathogens, the proportion of host individuals infected, or their infection titer, could decline. Any such reduction in host infection could in turn decrease host-to-tick transmission, resulting in lower tick infection prevalence. Although this mechanism is supported by theoretical models based on the Lyme disease system [16], it appears not to have been evaluated in field situations.

The results described here are a component of The Tick Project [6,17], which was an evaluation of whether two commercially available acaricidal products, deployed separately or together, reduced risk and incidence of tick-borne diseases in endemic areas. Previously, we reported that an acaricidal intervention targeted at ticks attached to small mammals, TCS (Tick Control System) bait boxes, significantly reduced abundance of host-seeking nymphal blacklegged ticks [6,18] compared to placebo controls. We also reported that a fungal biocide, Met52, consisting of the F52 strain of *Metarhizium brunneum*, did not significantly reduce tick abundance compared to placebo controls, whether used alone or in combination with TCS bait boxes. In this study, we tested the hypothesis that these two interventions would reduce infection prevalence of host-seeking blacklegged ticks with three tick-borne zoonotic pathogens, *B. burdgorferi*, *A. phagocytophilum*, and *B. microti*. We hypothesized that, by killing ticks on reservoir hosts, an acaricidal treatment targeted at small-mammal hosts (TCS bait boxes) would strongly reduce infection prevalence, whereas the areal biocide (Met52) aimed at questing ticks would have more modest effects on infection prevalence. We further hypothesized that any reductions in infection prevalence would strengthen through time.

## 2. Methods

### 2.1. Study Design

The Tick Project was a randomized, placebo-controlled, double-masked experiment undertaken in Dutchess County, New York, which is within the endemic zone of Lyme disease in the northeastern United States. Twenty-four residential neighborhoods were selected in 2016 for inclusion in the study, where each neighborhood consisted of roughly 100 homes and surrounding property. Approximately 34% of homeowners in each neighborhood elected to participate in the project. Average neighborhood size was 28 ha and average property size was 0.20 ha [6]. We randomly assigned neighborhoods to one of four treatment groups, which differed in the use of two acaricidal interventions and their corresponding placebo controls. The first intervention involved the use of MaxForce TCS bait boxes (hereafter TCS bait boxes, see Supplementary Material Figure S1), which are small, enclosed devices that attract small mammals and apply the chemical acaricide fipronil to them, effectively killing ticks on those hosts [19]. The small mammals themselves are unharmed by these devices. Active TCS bait boxes contained fipronil, while inactive bait boxes, acting as placebo controls, contained no fipronil but were otherwise identical. The second acaricidal intervention was the spraying of a water solution containing spores of Met52 (Novozymes), which is the F52 strain of the entomopathogenic fungus, *Metarhizium brunneum* [20,21]. The active Met52 intervention contained the fungus, while the placebo control for the Met52 intervention was plain water. Both products were used according to manufacturer recommendations. Both interventions and their respective controls were deployed each year from 2017 through 2020. Details regarding the timing and intensity of deployments are provided in Keesing et al. [6].

There were six replicates of each of four treatment groups: (1) active TCS bait boxes and active Met52; (2) active TCS bait boxes and inactive Met52; (3) inactive TCS bait boxes and active Met52; and (4) inactive TCS bait boxes and inactive Met52. The double-masked design meant that neither the members of participating households in the neighborhoods nor the researchers collecting data were aware of which treatment group was deployed in any neighborhood. Neighborhoods in the different treatment categories did not differ with respect to landscape composition or configuration, e.g., percent cover of forest vs. nonforest habitat [6].

### 2.2. Tick Collection

Questing ticks were collected in May–July of 2017, 2018, 2019, and 2021 (the COVID-19 pandemic prevented collection in 2020) from 20 randomly selected properties in each of the 24 neighborhoods (N = 480 properties). Collections in May–July were focused on the nymph stage of the blacklegged tick, which shows a seasonal peak in questing activity during this time [22]. We used flag-sampling (modified from [23]) with 1 m by 1 m squares of white corduroy cloth to collect ticks during timed intervals. Sampling was conducted in the three predominant habitat types in the study neighborhoods: forest, lawn, and shrub/garden. Ticks were removed from the cloth with forceps and maintained alive in humidified vials for later pathogen assays. Researchers collecting ticks were masked (blinded) to the treatment categories of the neighborhoods in which they were working.

### 2.3. Pathogen Detection

Within 2–3 weeks of being collected, ticks were surface-sterilized with 10% bleach, rinsed with deionized water, and then immediately stored individually in 2 mL Eppendorf tubes at −80 °C. Ticks were digested with Lysing Matrix I (MP Biomedicals, Irvine, CA, USA) and 5 μL DX antifoaming reagent ([24]; Qiagen) in 180 μL Buffer ATL and 20 μL proteinase K (Qiagen DNeasy 96 Blood & Tissue Kit) in Fastprep-24 (MP Biomedicals) for 1–3 min or with 3 mm tungsten beads in TissueLyser II (Qiagen) at 20 f/s for 4 min and incubated at 56 °C overnight in Buffer ATL and proteinase K. After 16–24 h of shaking in the incubator, the DNA was extracted using DNeasy 96 Blood & Tissue Kit (Qiagen, Maryland, USA) and stored in 4 °C or −30 °C for PCR analysis. Researchers assessing pathogen presence were masked (blinded) to the treatment categories of the neighborhoods from which the ticks were collected.

Using multiplex real-time PCR, as described in [14,25], *A. phagocytophilum* was detected by targeting the msp2 gene using primers ApMSP2f (59-ATG GAA GGT AGT GTT GGT TAT GGT ATT-39), ApMSP2r (59-TTG GTC TTG AAG CGC TCG TA-39), and ApMSP2p (5′-/5HEX/TGG TGC CAG/ZEN/GGT TGA GCT TGA GAT TG/3IABkFQ/-3′) (Integrated DNA Technologies). *B. burgdorferi* was detected by targeting the 23S ribosomal RNA (rRNA) gene using primers Bb23Sf (59-CGA GTC TTA AAA GGG CGA TTT AGT-39), Bb23Sr (59-GCT TCA GCC TGG CCA TAA ATA G-39), and Bb23Sp (5′-/56-FAM/AGA TGT GGT/ZEN/AGA CCC GAA GCC GAG TG/3IABkFQ/-3′).

Optimized conditions for multiplex PCR reactions were performed based on LightCycler 480 II (Roche) thermal cycler manufacturer recommendations. Briefly, PCR was performed in a multiplex format with a reaction volume of 20 μL by using 1X LightCycler 480 Probes Master (Roche), *A. phagocytophilum* and *B. burgdorferi* primers at 400 nM each, probes at 200 nM each (IDT), and 5 μL of template DNA. Tick larvae and PCR-grade water (Roche) were used as negative controls. All samples were tested in 3 replicates. Using Dual Color Hydrolysis Probe—UPL Probe 96-II program, the cycling conditions included an initial activation of the Taq DNA polymerase at 95 °C for 5 min, followed by 45 cycles of a 10 s denaturation at 95 °C, 30 s annealing at 51 °C, extension at 72 °C, and a 10 s cooling step at 40 °C.

*B. microti* infection was detected by targeting the 18S rRNA gene using primers smbaJF (5′-GCG TTC ATA AAA CGC AAG GAA GTG T-3′) and smbaKR (5′–TGT AAG ATT ACC CGG ACC CGA CG-3′) following a melt curve analysis [13]. SYBR Green I PCR reaction was performed based on manufacturer recommendations for the LC 480 II (Roche) thermal cycler by using LightCycler 480 SYBR Green I Master(Roche), JF and KR primers at 300 nM each, and 5 μL of template DNA in 20 μL reactions. Using SYBR Green I/HRM Dye program, the cycling conditions included an initial activation of the Taq DNA polymerase at 95 °C for 5 min, followed by 45 cycles of a 10 s denaturation at 95 °C, a 10 s annealing at 60 °C, and a 20 s extension step at 72 °C. Melting curve analysis was conducted at 97 °C with continuous 5 acquisitions per 1 °C, followed by a 30 s cooling step at 40 °C.

Positive controls for all three pathogens were taken from previous positive control samples [13,14] which were amplified and then cloned into *E. coli* using a TOPO^®^ TA kit and then cultured. Plasmid minipreps from liquid cultures were used to generate these positive controls which were verified by sequencing. All samples were run in three replicates.

Crossing point (Cp) values were obtained using the Absolute Quantification/Fit Point Analysis with the final cycle set to 40. The noiseband was adjusted above the background level, and the threshold was set to 1 for FAM and stayed as auto-threshold for HEX. To determine the Roche software efficiency in our *B. burgdorferi* final calls, we sent triturate and DNA extracts from 166 nymphal ticks to CDC laboratories [24], through which we confirmed that the parameters set in our analysis were ~95% similar. Samples that amplified in all three replicates and whose fluorescence was above that of the no-template control (NTC) and larvae were called positive. For *B. burgdorferi*, samples with rises for either one or two of the three replicates were considered positive if the amplification curves matched the exponential rise of the positive control and marginal if the curves had a horizontal rise, low fluorescence, or no rise but were *A. phagocytophilum*-positive. Marginal samples were rerun with *B. burgdorferi* singleplex and called positive if they amplified or negative if they did not. If samples amplified in all three replicates and their Cp values were within +/−1 of each other, they were called positive for *A. phagocytophilum*; otherwise, they were called marginal and tested with an *A. phagocytophilum*-confirmatory assay as described in [14]. Samples with rises for two out of three replicates were called marginal only if the amplification curves matched the exponential rise of the positive control, and the two values were within +/−1 of each other. We tested previously confirmed *A. phagocytophilum*-positive and *A. phagocytophilum*-negative samples [13] in Roche LC 480 II to determine the accuracy of our parameters and final calls, and our results matched 100%. In SYBR Green analysis, any replicates that amplified with a Cp < 37 and had a Tm = 83.5–84.8 were called positive for *B. microti*. Samples with double peaks, Tm = 83.0–83.5, or low fluorescence were called marginal and were retested [13].

### 2.4. Data Analysis

We analyzed infection data using R (version 4.0.1) [26] with the packages *tidyr* [27], *dplyr* [28], and *forcats* [29] for formatting and manipulating data and the package *ggplot2* [30] for graphing. We used the *broom.mixed* [31] package to tidy statistical data.

We analyzed infection data for each pathogen in two ways. In the first type of analysis, we included the first year of treatment (2017) as well as subsequent years (2018, 2019, 2021). In the second type of analysis, we included data for 2018–2021 only, excluding 2017, because of the expectation that TCS bait boxes and Met52 might not have had an effect on infection during the first year of treatment. For both types of analysis, we used linear mixed-effects models built with package *lme4* [32], log-transforming the data to conform to assumptions of tests. We treated neighborhood as a random effect and included year and an interaction between the presence of active TCS boxes and active Met52 treatments as fixed effects. For both types of analysis, we included only nymphal ticks and analyzed data from neighborhood-year combinations in which we were able to collect and assess infection for at least 10 nymphal ticks. The fit of models was evaluated using ANOVA following Satterthwaite’s method in package lmerTest [33].

## 3. Results

We assayed a total of 5380 nymphal *I. scapularis* ticks collected during 2017, 2018, 2019, and 2021. The overall mean proportion of nymphal ticks infected on control plots was 0.23 for *B. burgdorferi*, 0.14 for *A. phagocytophilum*, and 0.09 for *B. microti*.

**Figure 1:**
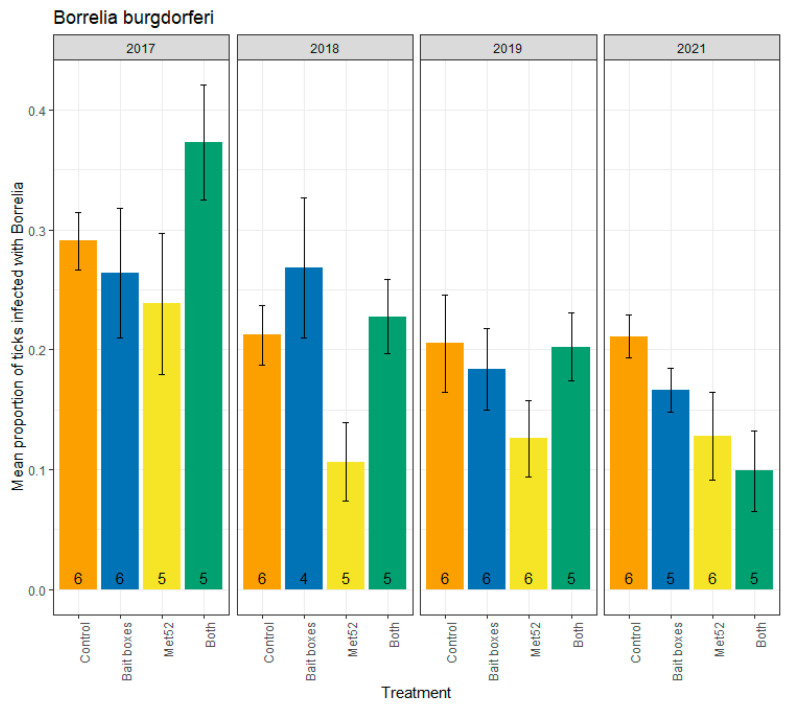
Mean proportion of blacklegged ticks, *Ixodes scapularis*, infected with *Borrelia burgdorferi*, as a function of the acaricidal treatment imposed on residential neighborhoods, over the four years ticks were sampled. Sampling did not occur in 2020 due to COVID-19 restrictions. On the x-axis, Control indicates neighborhoods that had placebo controls for both TCS bait boxes and Met52, Bait boxes indicates neighborhoods that had active bait boxes but placebo Met52, Met52 indicates neighborhoods that had active Met52 but placebo bait boxes, and Both indicates neighborhoods that had active TCS bait boxes and active Met52. Error bars are standard errors. Numbers on bars are the number of neighborhoods (out of 6) for which we were able to assess infection prevalence for at least 10 ticks.

The highest annual mean value for nymphal infection prevalence with *Borrelia burgdorferi* occurred in 2017 in neighborhoods assigned to the active TCS bait boxes and active Met52 treatment (0.37; N = 255 nymphs tested), and the lowest annual mean value occurred in 2021 in neighborhoods with the same treatment (0.10; N = 198 nymphs tested) (Figure 1). Contrary to our hypothesis, neighborhoods with active TCS bait boxes did not have lower infection prevalence with *B. burgdorferi* compared to placebo controls (F = 1.92, *p* = 0.18; Table 1).

The analysis based on all four years for which we collected ticks for testing (2017, 2018, 2019, and 2021) included the year of deployment (2017). We expected that the killing of immature ticks on small-mammal hosts in 2017 would not be likely to reduce infection prevalence of questing nymphs until the following year and thereafter. We therefore also ran analyses excluding the data from 2017. Again, we found that active TCS bait boxes were not associated with reduced *B. burgdorferi* infection prevalence (F = 1.27, *p* = 0.28; Table 1). In contrast, neighborhoods with active Met52 were associated with significantly lower infection prevalence with *B. burgdorferi* than the placebo controls (2017 removed, F = 8.21, *p* = 0.01; Table 1), or with a marginally nonsignificant change (2017 included, F = 3.39, *p* = 0.08; Table 1). Neighborhoods with both active TCS bait boxes and active Met52 were not significantly associated with reduced infection prevalence in the analysis with 2017 removed (F = 2.47, *p* = 0.13; Table 1), but analysis of all years revealed a significant reduction (F = 4.37, *p* = 0.05). Including all years in the analysis, we observed a significant effect of year (F = 21.81, *p* < 0.01); removal of 2017 from analyses resulted in a weaker effect (F = 3.63, *p* = 0.06). Inspection of the changes in infection prevalence with *B. burgdorferi* through the four years suggests a steady decrease through time (Figure 1), supporting our hypothesis of a cumulative effect of the interventions on *B. burgdorferi* infection prevalence.

**Figure 2:**
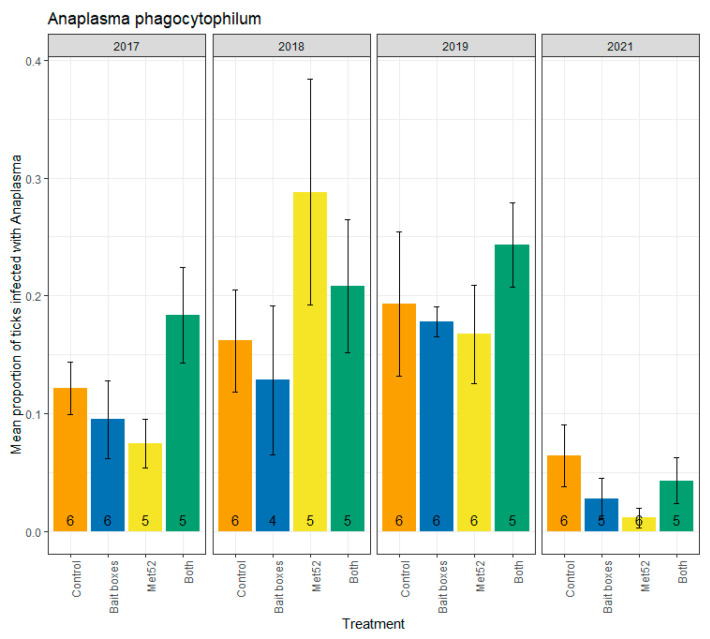
Mean proportion of blacklegged ticks, *Ixodes scapularis*, that were infected with *Anaplasma phagocytophilum*, as a function of the acaricidal treatment imposed on residential neighborhoods, over the four years ticks were sampled. Sampling did not occur in 2020 due to COVID-19 restrictions. On the x-axis, Control indicates neighborhoods that had placebo controls for both TCS bait boxes and Met52, Bait boxes indicates neighborhoods that had active bait boxes but placebo Met52, Met52 indicates neighborhoods that had active Met52 but placebo bait boxes, and Both indicates neighborhoods that had active TCS bait boxes and active Met52. Error bars are standard errors. Numbers on bars are the number of neighborhoods (out of 6) for which we were able to assess infection prevalence for at least 10 ticks.

Parallel analyses were run for infection prevalence with *A. phagocytophilum*, which ranged from a high of 0.24 (active Met52 neighborhoods and active TCS bait boxes in 2019) to a low of 0.02 (active Met52 neighborhoods in 2021) (Figure 2). Neither active TCS bait boxes, nor active Met52, nor both active interventions, were associated with reduced infection prevalence (Table 2). The lack of a significant effect of the active treatments on *A. phagocytophilum* infection prevalence was observed for analyses including and excluding ticks collected in 2017 (Table 2). For analyses including and excluding 2017, we observed a significant effect of year (Table 2). Although the F-values for year were significant (*p* < 0.01 for analyses with and without 2017; Table 2), inspection of Figure 2 suggests a unimodal pattern rather than a monotonic decline. Thus, the analyses of *A. phagocytophilum* infection support neither our hypothesis that TCS bait boxes would show the strongest impact nor the hypothesis that we would observe declines through time.

**Figure 3:**
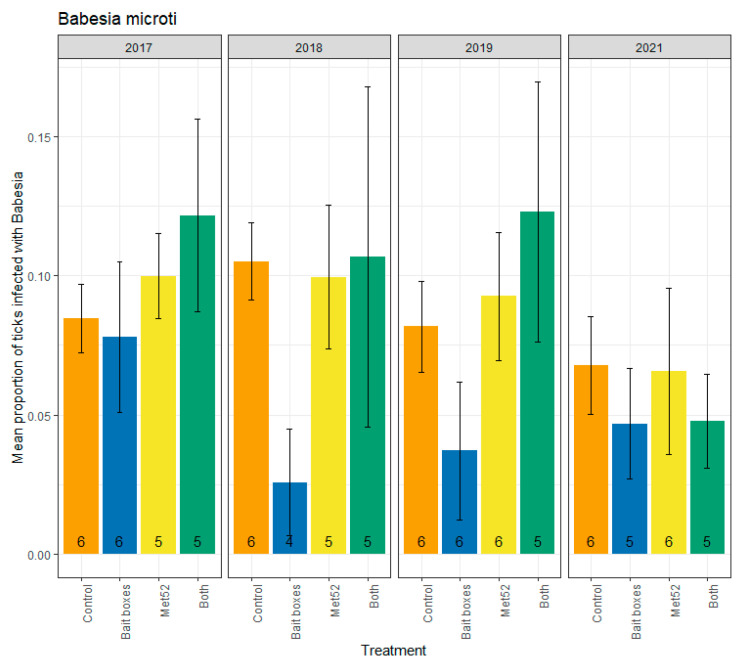
Mean proportion of blacklegged ticks, *Ixodes scaplaris*, that were infected with *Babesia microti*, as a function of the acaricidal treatment imposed on residential neighborhoods, over the four years ticks were sampled. Sampling did not occur in 2020 due to COVID-19 restrictions. On the x-axis, Control indicates neighborhoods that had placebo controls for both TCS bait boxes and Met52, Bait boxes indicates neighborhoods that had active bait boxes but placebo Met52, Met52 indicates neighborhoods that had active Met52 but placebo bait boxes, and Both indicates neighborhoods that had active TCS bait boxes and active Met52. Error bars are standard errors. Numbers on bars are the number of neighborhoods (out of 6) for which we were able to assess infection prevalence for at least 10 ticks.

Parallel analyses were run for infection prevalence with *B. microti*, which ranged from 0.14 (neighborhoods with both active treatments in 2019) to 0.04 (neighborhoods with active TCS bait boxes in 2018) (Figure 3). Whether the 2017 data were included or not, our analyses revealed no significant reduction in infection prevalence in neighborhoods with active TCS bait boxes, in neighborhoods with active Met52, or in those with both active treatments (Table 3). Therefore, our hypothesis that TCS bait boxes would have the strongest effect was not supported. We observed a significant effect of Year (F = 5.75, *p* = 0.02) in the analysis including 2017, but no significant effect of Year when 2017 was excluded (F = 2.22, *p* = 0.14). Although year was a significant determinant of infection prevalence when all years were included, an incremental decrease in infection prevalence was not apparent (Figure 3), failing to support our hypothesis.

## 4. Discussion

Although the primary purpose of acaricidal treatments is to reduce tick abundance, acaricides also have the apparent potential to reduce infection prevalence of ticks with zoonotic pathogens. In particular, acaricides targeted at the small-mammal hosts that act as primary reservoirs are designed to reduce both tick abundance and infection prevalence with tick-borne zoonotic pathogens [19]. Prior studies using the leading host-targeted acaricidal product, TCS bait boxes, report considerable efficacy at reducing abundance [34,35] or both abundance and infection prevalence of blacklegged ticks [19,34,36,37,38]. Acaricides targeted at host-seeking ticks, e.g., those sprayed in the environment, also can potentially reduce infection prevalence if diminished tick abundance disrupts tick-to-host and/or host-to-tick transmission [16].

Interventions using small-mammal targeted acaricides are often used in conjunction with area-wide chemical or biological acaricides, but typically not in a balanced design that would allow the separate and combined effects of the multiple interventions to be quantified. In addition, most studies do not include either experimental masking (blinding) or placebo controls. Placebo controls (e.g., [39]) and the masking of treatment designations (e.g., [40]) are deemed critical elements for minimizing various forms of implicit bias by researchers. The absence of balanced designs, of masking, and of placebo controls in these prior studies constrains our ability to directly compare our results to theirs. One other study that we are aware of used a randomized, placebo-controlled, masked design to evaluate the effects of TCS bait boxes on tick abundance and infection [41]. In their study of 622 households in Connecticut, Hinckley et al. [41] found no significant effect of active vs. placebo bait boxes on the prevalence of pathogen infection in blacklegged ticks with *B. burgdorferi*, *A. phagocytophilum*, or *B. microti*.

Our placebo-controlled, masked deployments of TCS bait boxes and Met52, separately and together, revealed no significant effect of TCS bait boxes on the prevalence of nymphal blacklegged ticks infected with *B. burgdorferi*, *A. phagocytophilum*, or *B. microti*. Small mammals, including white-footed mice, eastern chipmunks (*Tamias striatus*), short-tailed shrews (*Blarina brevicauda*), and masked shrews (*Sorex cinereus*), are the most competent reservoir hosts for all three of these pathogens [7,12,14,42]. However, the ability of other mammalian and avian hosts to transmit *B. burgdorferi* and *A. phagocytophilum* to larval blacklegged ticks is sometimes only modestly lower than those of small rodents and shrews [11,13,14]. In contrast, white-footed mice, eastern chipmunks, and shrews are considerably more competent reservoirs for *B. microti* than are any of the other mammalian and avian hosts tested [13]. Consequently, a stronger impact of acaricidal treatments targeted at these small mammal hosts might be expected for *B. microti*. Nevertheless, such effects of selectively targeting ticks on small mammals did not significantly reduce infection prevalence for any of these pathogens. Because shrews appear to be important reservoir hosts for a lineage of Powassan virus [43], which is also transmitted by blacklegged ticks, we recommend testing the efficacy of TCS bait boxes in reducing infection prevalence of this virus in questing ticks.

Nymphal blacklegged ticks in neighborhoods with active Met52 had significantly lower infection prevalence with *B. burgdorferi*, rejecting our hypothesis that Met52 would have more modest effects than TCS bait boxes, at least for this pathogen. However, for neither of the other pathogens did the active Met52 have a significant effect. In neighborhoods with both active TCS bait boxes and active Met52, infection prevalence with *B. burgdorferi* was significantly reduced compared to placebo controls, but for no other pathogen were such effects observed. For all three of the pathogens, we detected significant variation in infection prevalence through time. However, only for *B. burgdorferi* was there support for a monotonic decline from the beginning to the end of the study, and this pattern was observed across all treatments. Thus, for two of the three pathogens, our hypothesis of incrementally increasing effects of acaricidal treatments was not supported.

The effects of TCS bait boxes and Met52 on infection prevalence of questing nymphs are expected to accrue the year following deployment rather than in the same year. For TCS bait boxes, this is because any acaricidal effect on larval ticks attached to small mammals should affect infection prevalence of the resulting nymph stage, which becomes active the following year [19]. Similarly, the potential for Met52 to affect infection prevalence in questing nymphal ticks should require a year’s delay. This expectation arises because reduced tick abundance in the season of acaricidal deployment would disrupt tick-to-host and host-to-tick transmission in the same season, potentially resulting in reduced infection prevalence the following year. To account for the predicted one-year delay in the effect of both acaricidal treatments, we analyzed our data both including and excluding the initial year, 2017. Removal of the tick samples from 2017 had minimal impact on results for any of the three pathogens. We do note that, from 2018 to 2021, infection prevalence with *B. burgdorferi* remained consistent in the control neighborhoods, whereas in neighborhoods with active TCS bait boxes (with or without active Met52), prevalence steadily declined (Figure 1).

We previously reported [6] that neighborhoods with active TCS bait boxes, but not those with active Met52, had significantly lower abundance of blacklegged ticks than neighborhoods with placebo controls. We found no evidence for an interactive effect between the two treatments on tick abundance. Reduced prevalence of ticks infected with *B. burgdorferi* in the active Met52 neighborhoods, without reduced tick abundance, suggests the possibility of preferential killing of infected ticks. However, we are not aware of a mechanism that would cause this. We also reported [6] that the reduced abundance of questing nymphal blacklegged ticks, regardless of its cause, was not associated with reduced self-reported cases of tick-borne disease in human study participants. (In contrast, cases of tick-borne disease in outdoor pets were significantly lower.) The weak or nonexistent impact of the acaricidal interventions on infection prevalence for two of the three pathogens, reported here, may have weakened the potential for reduced tick abundance to prevent exposure to tick-borne pathogens and thus human disease.

In conclusion, our multiyear deployment of two tick-control interventions in residential neighborhoods within the endemic zone for tick-borne diseases in the northeastern United States revealed generally weak effects on infection prevalence of blacklegged ticks with tick-borne zoonotic pathogens. Exceptions were the reduced prevalence with *Borrelia burgdorferi* associated with the deployment of both active treatments together and the incremental reductions in *B. burgdorferi* infection prevalence throughout the four years of active TCS bait box treatments. Potentially, long-term deployments of these devices could enhance the direct tick-killing effects by also reducing prevalence of infection with *B. burgdorferi* and *Babesia microti* and provide a marginal additional benefit in reducing the probability of human exposure.

## Figures and Tables

**Table 1:** Results of statistical tests on infection prevalence of blacklegged ticks with *Borrelia burgdorferi*. We conducted two analyses of the data, one on all years of tick collections (i.e., including 2017, which was the year interventions were first deployed) and one excluding 2017 (i.e., only 2018–2021) using linear mixed effect models of log-transformed data, including only neighborhood-year combinations with at least 10 ticks tested. Abbreviations are: Sum Sq = sum of squares; NumDF = degrees of freedom, numerator; DenDF = degrees of freedom, denominator; F value = value of the F statistic; Pr(>F) = P value for the F statistic.

**2017–2021**	**Sum Sq**	**NumDF**	**DenDF**	**F Value**	**Pr(>F)**
Year	0.0213	1	61.95	21.81	0.00
BaitBox	0.0019	1	15.95	1.92	0.18
Met52	0.0033	1	15.93	3.39	0.08
BaitBox × Met52	0.0043	1	15.94	4.37	0.05
**2018–2021**	**Sum Sq**	**NumDF**	**DenDF**	**F value**	**Pr(>F)**
Year	0.0029	1	42.65	3.63	0.06
BaitBox	0.0010	1	18.12	1.27	0.28
Met52	0.0065	1	18.12	8.21	0.01
BaitBox × Met52	0.0019	1	18.15	2.47	0.13

**Table 2:** Results of statistical tests on infection prevalence of blacklegged ticks with *Anaplasma phagocytophilum*. We conducted two analyses, one on all years of tick collections (i.e., including 2017, which was the year interventions were first deployed) and one excluding 2017 (i.e., only 2018–2021) using linear mixed effect models of log-transformed data, including only neighborhood-year combinations with at least 10 ticks tested. Abbreviations are: Sum Sq = sum of squares; NumDF = degrees of freedom, numerator; DenDF = degrees of freedom, denominator; F value = value of the F statistic; Pr(>F) = P value for the F statistic.

**2017–2021**	**Sum Sq**	**NumDF**	**DenDF**	**F Value**	**Pr(>F)**
Year	0.5897	1	63	16.49	0.00
BaitBox	0.0044	1	16	0.12	0.73
Met52	0.0342	1	16	0.96	0.34
BaitBox × Met52	0.1129	1	16	3.16	0.09
**2018–2021**	**Sum Sq**	**NumDF**	**DenDF**	**F value**	**Pr(>F)**
Year	1.274822	1	60	42.43	0.00
BaitBox	0.000431	1	60	0.01	0.91
Met52	0.017934	1	60	0.60	0.44
BaitBox × Met52	0.029452	1	60	0.98	0.33

**Table 3:** Results of statistical tests on infection prevalence of blacklegged ticks with *Babesia microti*. We conducted two analyses, one on all years of tick collections (i.e., including 2017, which was the year interventions were first deployed) and one excluding 2017 (i.e., only 2018–2021), using linear mixed effect models of log-transformed data, including only neighborhood-year combinations with at least 10 ticks tested. Abbreviations are: Sum Sq = sum of squares; NumDF = degrees of freedom, numerator; DenDF = degrees of freedom, denominator; F value = value of the F statistic; Pr(>F) = P value for the F statistic.

**2017–2021**	**Sum Sq**	**NumDF**	**DenDF**	**F Value**	**Pr(>F)**
Year	0.1057	1	63	5.75	0.02
BaitBox	0.0518	1	17	2.82	0.11
Met52	0.0456	1	17	2.48	0.13
BaitBox × Met52	0.0453	1	17	2.47	0.13
**2018–2021**	**Sum Sq**	**NumDF**	**DenDF**	**F value**	**Pr(>F)**
Year	0.0428	1	40	2.22	0.14
BaitBox	0.0655	1	16	3.40	0.08
Met52	0.0310	1	16	1.61	0.22
BaitBox × Met52	0.0497	1	16	2.58	0.13

## Data Availability

Data will be made available in a public archive upon acceptance of the paper for publication.

## Supplementary Materials

The following supporting information can be downloaded at: https://www.mdpi.com/article/10.3390/pathogens12020172/s1, Figure S1: TCS bait box deployed in one of the study neighborhoods.

## References

[1] Ostfeld R.S., Price A., Hornbostel V.L., Benjamin M.A., Keesing F. (2006). Controlling ticks and tick-borne zoonoses with biological and chemical agents. Bioscience.

[2] Eisen L., Stafford K.C. (2021). Barriers to effective tick management and tick-bite prevention in the United States (Acari: Ixodidae). J. Med. Entomol..

[3] Sonenshine D.E., Mather T.N. (1994). Ecological Dynamics of Tick-Borne Zoonoses.

[4] Benjamin M.A., Zhioua E., Ostfeld R.S. (2002). Laboratory and field evaluation of the entomopathogenic fungus *Metarhizium anisopliae* (Deuteromycetes) for controlling questing adult *Ixodes scapularis* (Acari: Ixodidae). J. Med. Entomol..

[5] Hinckley A.F., Meek J.I., Ray J.A.E., Niesobecki S.A., Connally N.P., Feldman K.A., Jones E.H., Backenson P.B., White J.L., Lukacik G. (2016). Effectiveness of residential acaricides to prevent Lyme and other tick-borne diseases in humans. J. Infect. Dis..

[6] Keesing F., Mowry S., Bremer W., Duerr S., Evans A.S., Fischhoff I.R., Hinckley A.F., Hook S.A., Keating F., Pendleton J. (2022). Effects of tick-control interventions on tick abundance, human encounters with ticks, and incidence of tickborne diseases in residential neighborhoods, New York, USA. Emerg. Infect. Dis..

[7] Hersh M.H., Ostfeld R.S., McHenry D.J., Tibbetts M., Brunner J.L., Killilea M.E., LoGiudice K., Schmidt K.A., Keesing F. (2014). Co-infection of blacklegged ticks with *Babesia microti* and *Borrelia burgdorferi* is higher than expected and acquired from small mammal hosts. PLoS ONE.

[8] Prusinski M.A., Kokas J.E., Hukey K.T., Kogut S.J., Lee J., Backenson P.B. (2014). Prevalence of *borrelia burgdorferi* (spirochaetales: Spirochaetaceae), *anaplasma phagocytophilum* (rickettsiales: Anaplasmataceae), and *babesia microti* (piroplasmida: Babesiidae) in *ixodes scapularis* (acari: Ixodidae) collected from recreational lands in Hudson Valley Region, New York State. J. Med. Entomol..

[9] Hutchinson M.L., Strohecker M.D., Simmons T.W., Kyle A.D., Helwig M.W. (2015). Prevalence rates of *Borrelia burgdorferi* (Spirochaetales: Spirochaetaceae), *Anaplasma phagocytophilum* (Rickettsiales: Anaplasmataceae), and *Babesia microti* (Piroplasmida: Babesiidae) in host-seeking *Ixodes scapularis* (Acari: Ixodidae) from Pennsylvania. J. Med. Entomol..

[10] Ostfeld R.S., Keesing F. (2000). The function of biodiversity in the ecology of vector-borne zoonotic diseases. Can. J. Zool..

[11] LoGiudice K., Ostfeld R.S., Schmidt K.A., Keesing F. (2003). The ecology of infectious disease: Effects of host diversity and community composition on Lyme disease risk. Proc. Natl. Acad. Sci. USA.

[12] Keesing F., Brunner J., Duerr S., Killilea M., LoGiudice K., Schmidt K., Vuong H., Ostfeld R.S. (2009). Hosts as ecological traps for the vector of Lyme disease. Proc. R. Soc. B Biol. Sci..

[13] Hersh M.H., Tibbetts M., Strauss MOstfeld R.S., Keesing F. (2012). Reservoir competence of wildlife host species for *Babesia microti*. Emerg. Infect. Dis..

[14] Keesing F., Hersh M.H., Tibbetts M., McHenry D.J., Duerr S., Brunner J., Killilea M., LoGiudice K., Schmidt K.A., Ostfeld R.S. (2012). Reservoir competence of vertebrate hosts for *Anaplasma phagocytophilum*. Emerg. Infect. Dis..

[15] Levi T., Keesing F., Holt R.D., Barfield M., Ostfeld R.S. (2016). Quantifying dilution and amplification in a community of hosts for tick-borne pathogens. Ecol. Appl..

[16] Schauber E.M., Ostfeld R.S. (2002). Modeling the effects of reservoir competence decay and demographic turnover in Lyme disease ecology. Ecol. Appl..

[17] Keesing F., Ostfeld R.S. (2018). The Tick Project: Testing environmental methods of preventing tick-borne diseases. Trends Parasitol..

[18] Ostfeld R.S., Mowry S., Bremer W., Duerr S., Evans A.S., Jr Fischhoff I.R., Hinckley A.F., Hook S.A., Keating F., Pendleton J. (2023). Impacts over time of neighborhood-scale interventions to control ticks and tick-borne disease incidence. Vector-Borne Zoonotic Dis..

[19] Dolan M.C., Maupin G.O., Schneider B.S., Denatale C., Hamon N., Cole C., Zeidner N.S., Stafford K.C. (2004). Control of immature *Ixodes scapularis* (Acari: Ixodidae) on rodent reservoirs of *Borrelia burgdorferi* in a residential community of Southeastern Connecticut. J. Med. Entomol..

[20] Fischhoff I.R., Keesing F., Ostfeld R.S. (2017). The tick biocontrol agent *Metarhizium brunneum* (=M. anisopliae) (strain F52) does not reduce non-target arthropods. PLoS ONE.

[21] Bharadwaj A., Stafford K.C. (2010). Evaluation of *Metarhizium anisopliae* Strain F52 (Hypocreales: Clavicipitaceae) for control of *Ixodes scapularis* (Acari: Ixodidae). J. Med. Entomol..

[22] Ostfeld R.S., Levi T., Keesing F., Oggenfuss K., Canham C.D. (2018). Tick-borne disease risk in a forest food web. Ecology.

[23] Rulison E.L., Kuczaj I., Pang G., Hickling G.J., Tsao J.I., Ginsberg H.S. (2013). Flagging versus dragging as sampling methods for nymphal *Ixodes scapularis* (Acari: Ixodidae). J. Vector Ecol..

[24] Graham C.B., Maes S.E., Hojgaard A., Fleshman A.C., Sheldon S.W., Eisen R.J. (2018). A molecular algorithm to detect and differentiate human pathogens infecting *Ixodes scapularis* and *Ixodes pacificus* (Acari: Ixodidae). Ticks Tick. Borne. Dis..

[25] Courtney J.W., Kostelnik L.M., Zeidner N.S., Massung R.F. (2004). Multiplex real-time PCR for detection of *anaplasma phagocytophilum* and *Borrelia burgdorferi*. J. Clin. Microbiol..

[26] R Core Team (2022). R: A Language and Environment for Statistical Computing.

[27] Wickham H., Henry L. (2020). Tidyr: Tidy Messy Data.

[28] Wickham H., Francois R., Henry L., Muller K. (2020). Dplyr: A Grammar of Data Manipulation.

[29] Wickham H. (2020). Forcats: Tools for Working with Categorical Variables (Factors).

[30] Wickham H. (2016). Ggplot2: Elegant Graphics for Data Analysis.

[31] Bolker B., Robinson D. (2020). Broom.Mixed: Tidying Methods for Mixed Models.

[32] Bates D., Mächler M., Bolker B.M., Walker S.C. (2015). Fitting linear mixed-effects models using lme4. J. Stat. Softw..

[33] Kuznetsova A., Brockhoff P.B., Christensen R.H.B. (2017). lmerTest Package: Tests in linear mixed effects models. J. Stat. Softw..

[34] Schulze T.L., Jordan R.A., Williams M., Dolan M.C. (2017). Evaluation of the SELECT tick control system (TCS), a host-targeted bait box, to reduce exposure to *Ixodes scapularis* (Acari: Ixodidae) in a Lyme disease endemic area of New Jersey. J. Med. Entomol..

[35] Jordan R.A., Schulze T.L. (2019). Ability of two commercially available host-targeted technologies to reduce abundance of *Ixodes scapularis* (Acari: Ixodidae) in a residential landscape. J. Med. Entomol..

[36] Williams S.C., Little E.A.H., Stafford K.C., Molaei G., Linske M.A. (2018). Integrated control of juvenile Ixodes scapularis parasitizing *Peromyscus leucopus* in residential settings in Connecticut, United States. Ticks Tick. Borne. Dis..

[37] Williams S.C., Stafford K.C., Molaei G., Linske M.A. (2018). Integrated control of nymphal *Ixodes scapularis*: Effectiveness of white-tailed deer reduction, the entomopathogenic fungus *Metarhizium anisopliae*, and fipronil-based rodent bait boxes. Vector-Borne Zoonotic Dis..

[38] Little E.A.H., Williams S.C., Stafford K.C., Linske M.A., Molaei G. (2020). Evaluating the effectiveness of an integrated tick management approach on multiple pathogen infection in *Ixodes scapularis* questing nymphs and larvae parasitizing white-footed mice. Exp. Appl. Acarol..

[39] Kaptchuk T.J. (2001). The double-blind, randomized, placebo-controlled trial. J. Clin. Epidemiol..

[40] Grimes D.A., Schulz K.F. (2002). Bias and causal associations in observational research. Lancet.

[41] Hinckley A.F., Niesobecki S.A., Connally N.P., Hook S.A., Biggerstaff B.J., Horiuchi K.A., Hojgaard A., Mead P.S., Meek J.I. (2021). Prevention of Lyme and other tickborne diseases using a rodent-targeted approach: A randomized controlled trial in Connecticut. Zoonoses Public Health.

[42] Previtali M.A., Ostfeld R.S., Keesing F., Jolles A.E., Hanselmann R., Martin L.B. (2012). Relationship between pace of life and immune responses in wild rodents. Oikos.

[43] Goethert H.K., Mather T.N., Johnson R.W., Telford S.R. (2021). Incrimination of shrews as a reservoir for Powassan virus. Commun. Biol..

