# Supplementary figures and images for: Effects of Neighborhood-Scale Acaricidal Treatments on Infection Prevalence of Blacklegged Ticks (Ixodes scapularis) with Three Zoonotic Pathogens
Source: Pathogens. 2023 Jan 21;12(2):172. doi: 10.3390/pathogens12020172 (PMC9960617; doi:10.3390/pathogens12020172)

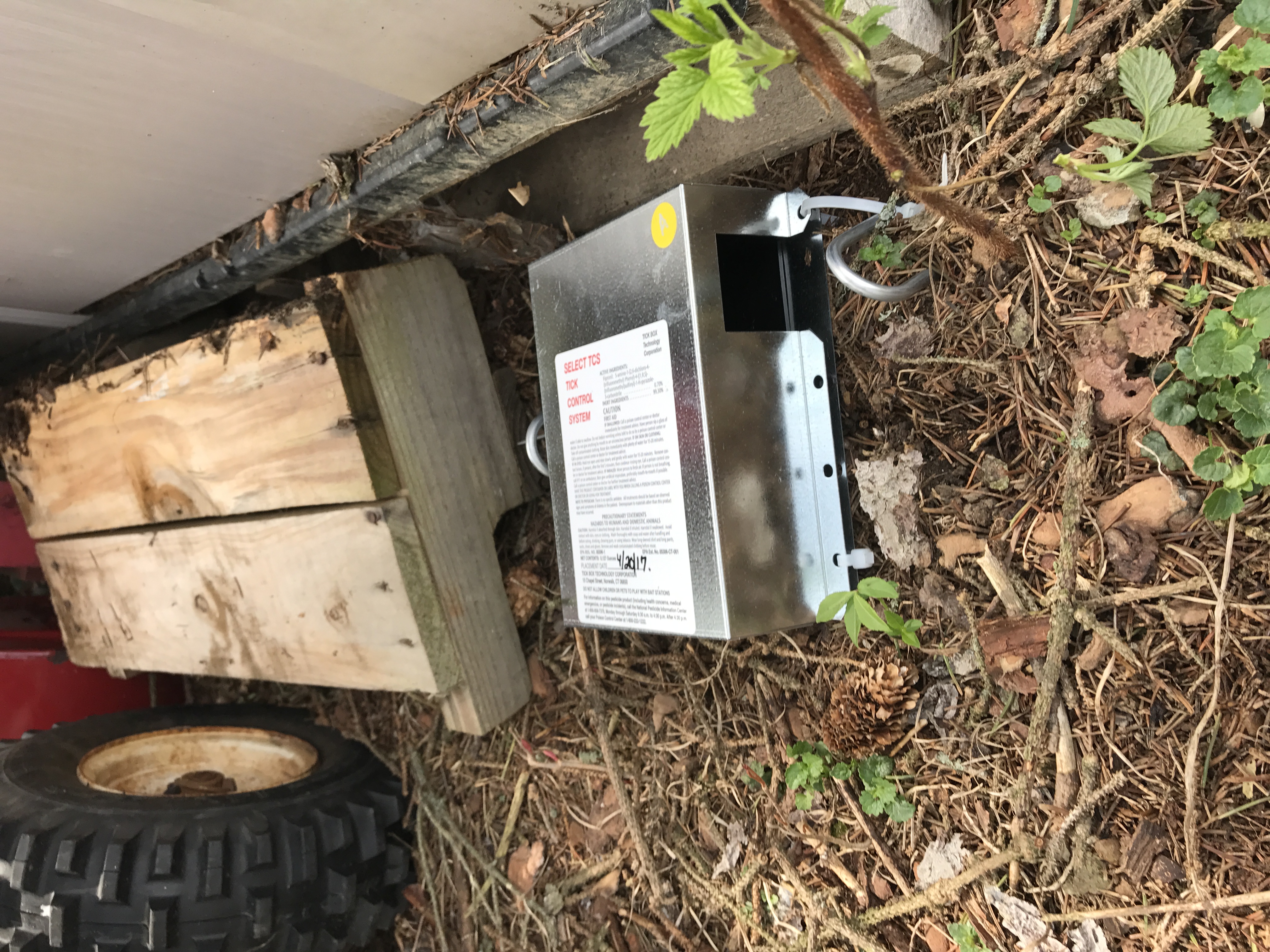

Supplement: Supplementary file 1 [file pathogens-12-00172-s001.zip › pathogens-2139613-supplementary.JPG]
